# Keep it positive: Exploring the relationship between stress, positive affect, wellbeing, and success of entrepreneurs

**DOI:** 10.3389/fpsyg.2022.970797

**Published:** 2022-09-28

**Authors:** Mateja Drnovšek, Alenka Slavec Gomezel

**Affiliations:** School of Economics and Business, University of Ljubljana, Ljubljana, Slovenia

**Keywords:** positive affect, stress, wellbeing, entrepreneur, success, mixed-method

## Abstract

Entrepreneurs’ wellbeing is of unprecedented importance given their crucial role in national economies in terms of job creation and innovation. In this research, we used a mixed methods approach to investigate the direct and indirect mechanisms by which entrepreneurs’ wellbeing mediates the effects of stress on perceived entrepreneurial success. We theorize that entrepreneurs experience work-related stress and that the level of perceived wellbeing mediates the relationship between the entrepreneurs’ stress and perceived success. We also hypothesize moderation effects by dispositional positive affect. We find that stress has direct negative effects on entrepreneurs’ wellbeing and perceived success. However, an experience of positive affect significantly weakens the negative effects of stress by broadening and building entrepreneurs’ cognitions. Drawing from our theoretical and empirical findings, we discuss implications for theory and practice in the domain of entrepreneurs’ wellbeing.

## Introduction

Workplace-related stress imposes significant costs upon national economies. 2020 officially became the most stressful year in recent history since 40% of all respondents across 115 countries experienced stress during a lot of the previous day according to the Gallup Institute (2022), and the COVID-19 pandemic has contributed additional challenges to the workforce (Nemteanu and Dabija, 2021; Priem, 2021). Stress-related diseases in their broadest sense contribute more to the total all-cause morbidity burden than cardiovascular disease (Kalisch et al., 2015). Moreover, stress appears to be greater for entrepreneurs than for other workers (Stephan et al., 2022). The experience of stress can lead entrepreneurs to develop negative coping skills, such as alcohol or other substance abuse, overeating, and social isolation (Dumont, 2016). When entrepreneurship is seen through the lens of behavioral addiction (Spivack et al., 2014), entrepreneurs express several negative physiological outcomes, such as obsessive thoughts, withdrawal, decreased self-worth, and neglect of previously important friends and activities (Spivack et al., 2014). In response to stress, entrepreneurs can develop active coping strategies to address the issues head-on (i.e., doing something to alter the stressful situation) or avoidance coping strategies (i.e., temporarily distancing themselves from a stressful situation). Although prior research in entrepreneurship has suggested that entrepreneurs can use both coping styles to deal with venture-related stress (Patzelt and Shepherd, 2011), the extant body of recent research has emphasized that to cope with stress and persevere psychological wellbeing, entrepreneurs may choose to devote less time to a business venture (Thomas and Ganster, 1995) or exit the venture altogether (Hsu et al., 2016).

While most research explored how entrepreneurs’ working conditions affect their mental and physical health (Gorgievski and Stephan, 2016 for a review), and their overall wellbeing, only a few studies have examined the activities within the personal sphere of entrepreneurs, such as positive affect, which plays the key role in living a healthy and meaningful life (Fredrickson, 2001). The importance of positive affect of entrepreneurs has gained credence in the literature ever since Baron (2008) explained its important role in the entrepreneurial process and proposed influences of positive affect on creativity, opportunity recognition, the ability to acquire needed resources, and the ability to respond quickly in dynamic environments. Among specific types of positive affect, the role of entrepreneurial passion has been studied in relation to entrepreneurial behaviors and various aspects of success (Cardon et al., 2009; Drnovsek et al., 2016; Murnieks et al., 2020b). However, significant questions on the role of positive affect for entrepreneurs remain. Specifically, within the experience of daily stressors, positive affect works as a facilitator of emotion regulation and reappraisal processes (Waugh, 2020), which are central stress resilience mechanisms (Kalisch et al., 2015). In entrepreneurship, business people often operate under conditions of high arousal and/or stress (Baron and Tang, 2011) which makes positive affect a critical cognitive resource for entrepreneurs, as it can buffer individuals against the affective costs of negative information (Baron et al., 2016). The experience of positive affect has been associated with enhanced cognitive flexibility due to increased dopamine levels (Ashby and Isen, 1999), and more effective decision-making (Lăzăroiu et al., 2017).

In this research, we examine the direct and indirect mechanisms by which perceived stress relates to entrepreneurial success. In entrepreneurship, exposure to daily stress has been identified as one of the main factors leading to founders’ exhaustion (Murnieks et al., 2020a), and a negative perception of success as an entrepreneur. We consider entrepreneurs’ job satisfaction to be a proxy of an entrepreneur’s overall wellbeing (Cooper and Artz, 1995; Binder and Coad, 2013; Wach et al., 2016). Given the struggles that entrepreneurs face in their daily lives (Nambisan and Baron, 2013), we emphasize the important role of positive emotions in helping entrepreneurs to persist in the face of difficulties (Cardon et al., 2012) to explore whether positive affect moderates the relationship between perceived stress and an entrepreneur’s wellbeing. In so doing, we add to the literature on entrepreneurial wellbeing and success (Parasuraman et al., 1996; Walker and Brown, 2004; Wach et al., 2016; Uy et al., 2017; Wiklund et al., 2019).

**FIGURE 1:**
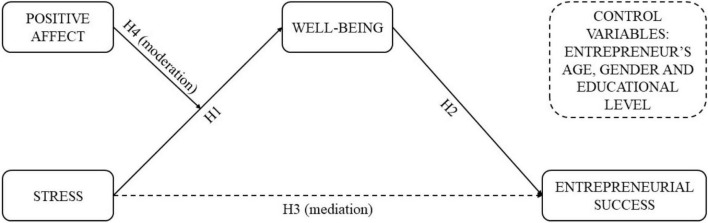
Conceptual model.

The findings from our study make an important contribution to the existing knowledge on the individual-level determinants of wellbeing in entrepreneurship (Parasuraman et al., 1996; Walker and Brown, 2004; Wach et al., 2016). With this study, we also illuminate the important role played by stress and its continuous presence in entrepreneurial workplaces. We emphasize the role of positive affect in moderating an entrepreneur’s capacity to manage stress. Our model of moderated-mediation relationships is presented in Figure 1. In the model, we explain how the intensity of perceived stress is associated with an entrepreneur’s wellbeing by postulating the direct effects of stress on the level of perceived entrepreneurial success and positive interaction of dispositional positive affect.

## Entrepreneurs’ wellbeing and stress

### Stress in entrepreneurship

Despite advances in technology and industrialization, the demands of occupational life have increased significantly over the last decades (Schieman et al., 2009; Schaufeli and Taris, 2014). Across occupations, the level of demand is particularly high in entrepreneurship (Cardon and Patel, 2015), since entrepreneurs work long hours in unpredictable, ever-changing work environments (Morris et al., 2015). Furthermore, they must take full responsibility for the consequences of their decisions (Buttner, 1992).

Overall, when creating and running their businesses, entrepreneurs are exposed to conditions that are known to generate high levels of work-related stress (Stephan and Roesler, 2010; Cardon and Patel, 2015; Torrès and Thurik, 2018). Work-related stress can be seen as a particular relationship between an individual and his/her work environment, in which the requirements of the work-related roles exceed the individual’s capabilities and accessible resources, are perceived as threatening to one’s wellbeing (Folkman et al., 1986); and require psychological, physiological, and behavioral efforts to exert control over the event and its outcomes (Lazarus and Folkman, 1984). The level of work-related stress experienced is a subjective response to threatening workplace conditions and is dependent upon the entrepreneur’s interpretation of his or her access to and control over mental and physical resources (Leka et al., 2004).

Entrepreneurial workplaces are full of stressors—stress-triggering events and causes (Harris et al., 1999) that are induced by working long hours (Grant and Ferris, 2012); engaging in multiple role identities such as inventor, founder, and developer (Cardon et al., 2009), which are associated with performing different entrepreneurial tasks—opportunity recognition, resource acquisition, and decision-making in uncertain and ambiguous situations (Douglas and Shepherd, 2000) not having enough time to complete work tasks, and get enough rest (Patterson et al., 2005). In addition, entrepreneurs remain fully accountable for their businesses and employees (Parasuraman and Simmers, 2001), and to compete in highly competitive and ever-changing environments (Kariv, 2008), they often operate under severe financial constraints (Fraser et al., 2015). On the other hand, entrepreneurs do have some advantageous resources that can help them cope with stress, such as autonomy and job control (Tetrick et al., 2000; Cardon and Patel, 2015), and their stress experiences are independent of whether the firm is performing well (Gumpert and Boyd, 1984). This suggests that it is the entrepreneurs’ overall daily tasks and challenges that likely induce stress (Hessels et al., 2017). Expectancy theory provides the fundamental logic in terms of how perceived stress may be associated with success through the mediation of individual-level processes, such as perceived wellbeing. Expectancy theory includes two mechanisms: the level of demand expectancy and the demand valence mechanism. Therefore, different types of stressors are likely to be associated with entrepreneurs’ beliefs regarding the relationship between the level of effort invested in coping with the demands at hand and the probability of success in meeting those demands (LePine et al., 2005).

### Entrepreneurs’ wellbeing as a key outcome in entrepreneurship research

The vast majority of prior research in entrepreneurship has measured entrepreneurial outcomes in terms of financial performance indicators, such as sales, growth, and profits (Lumpkin and Dess, 2001; Akgüna et al., 2007; Baron et al., 2011). In a recent review Shepherd et al. (2019) identified that firm performance was a dominant dependent variable in entrepreneurship research. Acknowledging that there are various motivations for pursuing an entrepreneurial career, with financial motives being only one of them, other dependent variables need to be considered. The extant entrepreneurial literature concurs that entrepreneurs’ wellbeing is an important aspect of non-monetary success (Parasuraman et al., 1996; Walker and Brown, 2004; Wach et al., 2016; Uy et al., 2017). Wellbeing has been recognized as an important variable in entrepreneurship, as it is an integral part of living a fulfilling and flourishing life, and entrepreneurship can be a source of personal fulfillment and satisfaction (Cooper and Artz, 1995; Wiklund et al., 2019). Entrepreneurs’ wellbeing has been associated with a range of positive outcomes in entrepreneurship at both the individual and firm levels (Stephan, 2018), such as opportunity identification, creativity, and risk-taking (Nikolaev et al., 2020). Many individuals engage in entrepreneurship because they aspire to achieve greater professional career satisfaction through autonomy and independence, rather than being employed by others (Naffziger et al., 1994; Walker and Brown, 2004). They also desire to free themselves from the social structures and conditions they find restrictive (Rindova et al., 2009; Wach et al., 2016). In this study we see entrepreneurs’ wellbeing as job-satisfaction in running an entrepreneurial venture, which is a view aligned with current discussions in the entrepreneurship literature (Nikolaev et al., 2019; Wiklund et al., 2019). Entrepreneurship research has early emphasized the importance of job satisfaction for entrepreneurs (Cromie and Hayes, 1991; Cooper and Artz, 1995; Srivastava et al., 2001; Baron et al., 2016) since it impacts an entrepreneur’s future decision-making with respect to investing more personal finance and sweat equity (Smilor, 1997) and/or exiting or closing down the firm. Wach et al. (2016) showed that entrepreneurial success is captured by personal fulfillment in terms of the degree of happiness associated with job and work engagement. Indeed, prior research has suggested that for entrepreneurs, wellbeing is more closely related to satisfaction with life, self, and family than it is for employees. This finding reflects the centrality of work in an entrepreneur’s life (Loewe et al., 2015) and sources of an entrepreneur’s identity (Murnieks et al., 2020b).

## Hypotheses

### The direct relationship between perceived stress and wellbeing of entrepreneurs

Prior evidence suggests negative effects of stress on entrepreneurs’ experience of subjective wellbeing (Srivastava et al., 2001; Baron et al., 2016; Lerman et al., 2020). Stress is primarily an emotional experience that is associated with nervousness, tension, and strain (Cooke and Rousseau, 1984; Van Dyne et al., 2002), and is expressed as felt job stress (Motowidlo et al., 1986). Felt job stress refers to a sense of time pressure, anxiety, and worry associated with a job that needs to be done (Hunter and Thatcher, 2007; Lerman et al., 2020). High levels of felt job stress have been associated with low job satisfaction (Terry et al., 1993), burnout, and low overall life satisfaction (Ganster and Schaubroeck, 1991). Entrepreneurs who are exposed to high levels of work-related stress are expected to experience low task performance and negative work-related attitudes (Hunter and Thatcher, 2007; White and Gupta, 2020). Because work-related stress has a direct impact on the physical and emotional aspects of an entrepreneur’s health, it can take a significant toll that manifests itself in substantial decreases in personal wellbeing (Sonnentag, 2001). Thus, we postulate a negative relationship between the level of perceived stress and the level of perceived wellbeing of the entrepreneur.

**Hypothesis 1:** An entrepreneur’s level of perceived stress will be associated with a lower level of perceived wellbeing.

### The direct relationship between wellbeing and entrepreneurial success

Research has shown that achieving satisfactory wellbeing in the workforce is an important priority (Grant et al., 2007), and wellbeing in entrepreneurial performance is widely acknowledged (Dijkhuizen et al., 2018; Nikolaev et al., 2020). In work psychology literature several mechanisms have been used to explain why workers who experience higher levels of wellbeing perform better (Bakker and Demerouti, 2007) which in the case of entrepreneurs can be expected to relate to entrepreneurial success and better business performance (Dijkhuizen et al., 2018). The first mechanism refers to the role of positive emotions in broadening peoples’ “thought-action repertoires” which results in enhanced personal resources (Fredrickson and Branigan, 2005). Second, entrepreneurs who experience higher wellbeing will likely experience better health (Friedman and Kern, 2014; Wiklund et al., 2019), directing their productive energy to their work and the subsequent experience of success. This leads us to propose the following:

**Hypothesis 2:** An entrepreneur’s level of perceived wellbeing will be associated with a higher level of entrepreneurial success.

### The indirect relationship between perceived stress and entrepreneurial success

As prior research in entrepreneurship tells us that entrepreneurs’ job-related stress can critically impact business success and survival (Morris et al., 2015), exit from entrepreneurship (Baron et al., 2016), and the individual’s capability to recover through sleep and wellbeing (Kollmann et al., 2019) we also postulate an indirect effect of stress on entrepreneurial success. To conceptualize the mediating role of wellbeing in the relationship between perceived stress and entrepreneurial success, we return to the argument supporting a direct relationship between stress and wellbeing (H1). The main idea is that perceived stress harms wellbeing due to an increased instance of negative emotional experiences, such as nervousness, anxiety, tension, and strain that entrepreneurs experience. Joining this with the argument in support of a direct relationship between wellbeing and entrepreneurial success (H2), we argue that stress deteriorates the level of perceived entrepreneurial success by decreasing entrepreneurs’ wellbeing. Drawing from the above discussion, we argue that there is an indirect relationship between the entrepreneur’s level of perceived stress and his/her perceived entrepreneurial success because stress is negatively associated with the level of entrepreneurial success, and stress is negatively related to the level of perceived wellbeing. This leads us to propose:

**Hypothesis 3**: Wellbeing mediates the negative relationship between an entrepreneur’s level of stress and entrepreneurial success.

### The moderating role of positive affect

Dispositional positive affect refers to a stable tendency to experience positive moods and emotions (Baron et al., 2011). Individuals with high dispositional positive affect tend to perceive things through »pink lens« while people with high negative affectivity tend to perceive things through »black lens«, an effect which has immediate impact on individuals’ sensations and behaviors (Barsade and Gibson, 2007). Moreover, prior research emphasizes »positivity« in organizational life (Luthans, 2002). Similarly, entrepreneurship scholars have emphasized the important role that affect plays in explaining entrepreneurial behaviors and outcomes (Baron, 2008; Foo et al., 2009; Baron et al., 2012; Cardon et al., 2012).

We expect that the entrepreneur’s positive affect will moderate the direct effects of stress on his/her wellbeing such that this relationship will be stronger for entrepreneurs who experience higher positive affect. In the entrepreneurship literature, dispositional positive affect has been defined as “stable tendencies to experience positive affect often, and across many situations” (Baron and Tang, 2011, p. 4)—a baseline to which individuals tend to return. Positive affect is thus a stable individual difference in one’s affective tone (Kaplan et al., 2009).

Positive affect serves as a stress-buffering resource by influencing stress-sensitive metabolic hormones (Moskowitz et al., 2008; Cardon and Patel, 2015). Positive affect directs entrepreneurs to focus their attention on aspects of the situation that are congruent with their mood (Baron, 2008), improved performance (Kaplan et al., 2009), and subjective wellbeing (Lyubomirsky et al., 2005; Lyubomirsky and Layous, 2013). Foo et al. (2009) found that positive affect increases subsequent effort on venture tasks beyond what is immediately required. Furthermore, Baron and Tang (2011) found that entrepreneurs have a higher positive dispositional affect than other employment groups. For these reasons, positive dispositional affect is particularly important for understanding entrepreneurial wellbeing (Cardon and Patel, 2015). Finally, positive affect provides individuals with information that progress is being made toward their goals (Carver and Scheier, 2014). Therefore, positive affect should weaken the direct effects of stress on the level of perceived wellbeing. This leads us to propose:

**Hypothesis 4**: The positive affect of an entrepreneur moderates the direct relationship between stress and his/her wellbeing in such a way that the effects of the perceived level of stress are weaker for higher levels of positive affect.

## Materials and methods

### Research design and sample

We used a mixed method explanatory sequential research design (Molina-Azorín et al., 2012; Creswell, 2015). We followed a two-phase design in which we initially collected quantitative data, followed by qualitative data. The purpose of the qualitative data collection was to further explain and interpret our findings from the quantitative data (Plano Clark and Ivankova, 2016), and to increase the validity of our study by converging and corroborating the results from different methods (Creswell, 2015). In our study the respondents from the qualitative data collection differed from the respondents in the quantitative data collection. Overall, the mixed methods design for our data collection (survey and interviews) and data analysis (statistical analysis and content analysis) provided a more detailed investigation of the phenomena under study, especially given the increasing interest in the topic to date (Creswell, 2015).

In Study 1, we collected quantitative data through an online survey among small business owners in Slovenia. In May 2018, we sent the survey link to 1,700 entrepreneurs of small firms (up to 49 employees), which were randomly selected from the database Bizi.si, which provides up-to-date financial and contact information of Slovenian firms. In Bizi.si, we restricted sampling to firms with up to 49 employees and extracted those contacts for which an email address was provided by Bizi.si. After excluding several contacts for which email checks provided an unsuccessful reach, we retained 1,700 firms in the sample. We received 279 responses yielding a 16% response rate. After excluding 57 surveys that were not completed fully, we continued with a sample of 222 responses. Our first question in the survey was a filter question: “Are you a founding entrepreneur of the firm?” Survey respondents who chose “No” were excluded from the analysis. The final data sample included 152 respondents who had completed the survey in full, i.e., 54% usable responses out of the 279 responses received.

In the final sample, 53% of the respondents were women and 47% were men; the youngest respondent was 22 years old and the oldest was 68 years old, with a mean age of 44 years (SD = 11.7). In terms of their entrepreneurial experience, 86% of respondents stated that their current business was their first entrepreneurial venture. The education level of responding entrepreneurs was high, with 48% of entrepreneurs having a college education or higher. When asked about the size of their business, 65% of respondents indicated that they had up to five employees, 23% of respondents indicated that they had between 6 and 10 employees, and 5% indicated that they employed between 11 and 15 employees. The rest of the respondents (7%) employed 15–49 employees. A large majority of the respondents (78%) indicated that business services were their primary industry, 4% stated production as their primary industry, and 18% reported that their business offered a combination of services and products.

In Study 2, we used a qualitative methodological approach. From March to May 2019, we conducted in-depth personal interviews using open-ended questions with 15 entrepreneurs (11 respondents were male—73%) from different industries (based on the Standard Classification of Activities, the three most represented industries were G = wholesale and retail trade, repair of motor vehicles and motorcycles, M = professional, scientific and technical activities, and J = information and communication). Respondents ran firms of different sizes (five firms were micro firms with up to nine employees, and 10 firms were small firms with up to 49 employees). Respondents indicated different performance results, with two firms experiencing a decrease in revenue from 2017 to 2018, three firms maintaining their level of revenue, seven firms reporting a moderate increase in revenue, and three firms experiencing a remarkable increase in revenue from 2017. We recorded and transcribed all interviews. The qualitative content analysis of the text was done manually—the coding process was done by one of the authors of this research in two phases—first, the codes from the interviewees’ quotes were formed, and second—the themes from the first-phase codes were formed. Then, the codes and themes were reviewed by the third person. There were only minor discrepancies in the interpretation of codes, which were adjusted.

### Measures

*Perceived stress* was measured using the Perceived Stress Scale (Cohen et al., 1983), which measures the degree to which situations in one’s life are perceived as stressful. Items are designed to assess how unpredictable, uncontrollable, or overloaded respondents find their lives to be. Respondents rated how often they felt or thought in the way described by the items, using a 5-point Likert scale ranging from “Never” to “Very often.” A sample item was: “In the last month, how often have you been upset because of something that happened unexpectedly?” The item “In the last month, how often have you felt nervous and ‘stressed’?” did not load properly and was excluded from the analysis. The reliability of the 9-item measurement scale was good (Cronbach’s alpha = 0.867).

*Positive affect* was measured using the Positive and Negative Affect Schedule (PANAS) (Watson et al., 1988), which includes high-activation forms of positive affect. Positive affect was assessed by our respondents with 10 items on a 5-point Likert scale. We asked them to rate how often in general they experienced the described feelings and emotions, such as “strong” and “interested.” Four items “inspired,” “excited,” “alert,” and “active” did not load properly on the intended factor or cross-loaded and so were excluded from further analyses. PANAS assesses positive affect as an individual state, not as a personal disposition (George and Zhou, 2002). The reliability of the 6-item measurement scale was good (Cronbach’s alpha = 0.806).

We measured entrepreneurs’ *wellbeing* as job satisfaction, which was assessed using a 5-item Brief Job Satisfaction Measure II (Judge et al., 1998), which includes statements regarding respondents’ experience at work. This measurement approach is aligned with recommendations from recent work on wellbeing in entrepreneurship (Wiklund et al., 2019). Small business owners rated their level of agreement on a 7-point Likert scale. A sample item was: “I feel fairly well satisfied with my present job.” The reverse-coded items “Each day of work seems like it will never end” and “I consider my job rather unpleasant” did not load properly, so we excluded them from further analysis. The reliability of the measure based on Cronbach’s alpha was 0.835.

We measured *entrepreneurial success* as an index in which we averaged the reported firm success in the last 3 years in terms of revenue growth (1 = much worse than competitors, 5 = much better than competitors) and market share growth (1 = much worse than competitors, 5 = much better than competitors) and divided this score by the geographical position of customers (1 = mostly foreign markets, 2 = mostly national market, 3 = mostly local market), resulting in a relative measure of entrepreneurial success. In so doing, firms with reported high scores on revenue growth and market share growth locally got a lower score than those who reported a high score on revenue growth and market share growth internationally. All measures were taken from the survey in which we asked respondents to rate their success in terms of revenue growth and market share growth in the last 3 years in comparison to their competitors and to select where majority of their customers came from.

We controlled for the entrepreneur’s gender, age, and level of education. Table 1 presents the correlations among the studied variables, along with the descriptive statistics and square roots of the average variance explained. In Appendix Table 1, we present the final scale items and their item loadings based on confirmatory factor analysis.

**TABLE 1:** Descriptive statistics, correlations and square roots of the average variance explained.

Variables	Mean	SD	1.	2.	3.	4.	5.	6.
1. Stress	2.560	0.581	0.811					
2. Wellbeing	5.649	1.119	−0.452***	0.893				
3. Positive affect	3.837	0.560	−0.436***	0.462***	0.889			
4. Success	3.464	1.491	−0.317***	0.407***	0.283***	/		
5. Gender	0.533	0.501	0.171*	0.171	0.351***	0.039	/	
6. Age	44.130	11.540	–0.125	–0.086	−0.307***	–0.150	−0.384***	/
7. Educational level	3.574	0.781	–0.048	0.195*	0.204*	0.196*	0.216**	−0.250***

*N* = 152; *p < 0.05; **p < 0.01; ***p < 0.001.

All effects are two-tailed tests. Square roots of average variance explained are on the diagonal.

To reduce the potential for common method variance, we applied procedural and statistical remedies *ex ante* and *ex post* data collection and data analysis based on the recommendations of Richardson et al. (2009) and Podsakoff et al. (2012). In terms of *ex ante* remedies, we assured respondents’ anonymity, explained the purpose of the study in the e-mail containing the link to the survey, pre-tested the instructions, items, and layout of the survey, labeled all scale points, and used different scale types and anchor labels. The results were analyzed using different techniques. First, the common method factor based on a confirmatory factor analysis examination evidenced a 0.00% common variance. Second, the total variance explained based on the factors with eigenvalues greater than 1.0 was 25.29%. We checked the variance inflation factor (VIF) among the studied variables, with the highest VIF being between positive affect and entrepreneurial success (VIF = 1.591). This value is well below the problematic threshold of 10 (Neter et al., 1990) or even the more stringent threshold value of 5 or 2.5 (Allison, 2012). This suggests that multicollinearity does not pose a threat to our data. Furthermore, our model incorporates a moderation effect. Hypotheses about interaction effects are less likely to be subject to common method bias because respondents are less likely to recognize the relationships among studied variables if there is a moderator in the theoretical model (Aiken and West, 1991; Harrison et al., 1996). This suggests that including a moderator variable to the theoretical model adds to the cognitive effort that respondents would need to presume the nature of the relationships under investigation by the researchers. Although we cannot be completely certain that common method variance is absent from our study, the reported remedies and tests suggest that it is not a threat to the data in this research.

### Statistical analyses

We ran confirmatory factor analysis on the measurement and structural model. In examining our final measurement model, we used the following goodness of fit indices: CFI (comparative fit index), RMSEA (root mean square approximation), and SRMR (standardized root mean square residual). The values of the fit indices of the final model before hypotheses testing with correlations among the variables under study were the following: χ^2^ = 287.160; df = 190; *p* = 0.000; CFI = 0.920; RMSEA = 0.058; SRMR = 0.077. After establishing a good fit of the latent and control variables to the data, we proceeded with mean centering of the latent variables and reducing the scales into single index variables to conduct the mediation-moderation analysis in the structural model. The model fit of the moderated-mediation model was good: χ^2^ = 11.585; df = 11; *p* = 0.396; CFI = 0.996; RMSEA = 0.019; SRMR = 0.055. For all reported statistical analyses, we used either IBM SPSS 21.0 or IBM AMOS 20.0 software.

## Results

### Study 1: Quantitative data

We tested the mediation-moderation theoretical model in several ways. For the mediation analysis, we followed the mediation analysis steps laid out by Rucker et al. (2011) and Hayes (2017). We were interested in the significance of the indirect effects and the effect sizes accompanying those effects. The moderation analysis was performed by introducing the interaction effect into the mediation model and analyzing the interaction effect (Aiken and West, 1991).

**FIGURE 2:**
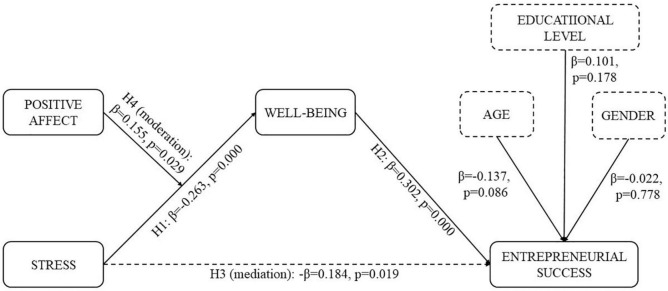
Results of model testing.

Hypothesis 1 predicted a negative association between entrepreneurs’ perceived stress and the level of their perceived wellbeing. The results reported in Figure 2 confirm hypothesis 1 (β = −0.263, *p* < 0.001) as has been shown in previous research (Srivastava et al., 2001; Baron et al., 2016). Hypothesis 2 suggested a positive association between perceived wellbeing and entrepreneurial success and the hypothesis was confirmed by the results of our study (β = 0.302, *p* < 0.001). With these results, we corroborate the acknowledged importance of wellbeing for entrepreneurial performance (Dijkhuizen et al., 2018) by enhancing personal resources (Fredrickson and Branigan, 2005) and experiencing better health and productive energy for their entrepreneurial tasks (Friedman and Kern, 2014).

Next, to test Hypothesis 3 we analyzed the mediation effect of perceived wellbeing on the relationship between perceived stress and entrepreneurial success utilizing the bootstrapping method. The results reported in Table 2 show that perceived wellbeing partially mediated this relationship (indirect effect: β = −0.083, *p* = 0.005, −0.167 < bootstrap confidence interval < −0.027). However, there was also a statistically significant direct relationship between perceived stress and entrepreneurial success (β = −0.185, *p* = 0.009). The results provide support for Hypothesis 3 that perceived wellbeing partially mediates the relationship between perceived stress and entrepreneurial success. In prior entrepreneurial research such mediational relationship was not directly tested, yet research showed that stress could critically impact business survival (Morris et al., 2015), entrepreneurial exit (Baron et al., 2016) and recovery through sleep and wellbeing (Kollmann et al., 2019). Thus, we provide a better understanding of the relationship between an entrepreneur’s perceived stress and entrepreneurial success through subjective wellbeing.

**TABLE 2:** Results for the decomposition of effects in the moderated mediation model using a bootstrap method.

	Wellbeing				Entrepreneurial success			
		
	Unstd. coefficient	Std. error	Std. coefficient	*P*-value (two-tailed significance)	Unstd. coefficient	Std. error	Std. coefficient	*P*-value (two-tailed significance)
**Stress**								
Direct effect	–0.265	0.102	–0.263	0.009	–0.186	0.073	–0.185	0.009
Indirect effect	0.000	0.000	0.000	−	–0.084	0.031	–0.083	0.005
Total effect	–0.265	0.102	–0.263	0.009	–0.270	0.077	–0.268	0.000
**Positive affect**								
Direct effect	0.270	0.071	0.278	0.000	0.000	0.000	0.000	−
Indirect effect	0.000	0.000	0.000	−	0.086	0.031	0.088	0.000
Total effect	0.270	0.071	0.278	0.000	0.086	0.031	0.088	0.000
**Wellbeing**								
Direct effect	−	−	−	−	0.317	0.067	0.317	0.000
Indirect effect	−	−	−	−	0.000	0.000	0.000	−
Total effect	−	−	−	−	0.317	0.067	0.317	0.000

*N* = 152.

**FIGURE 3:**
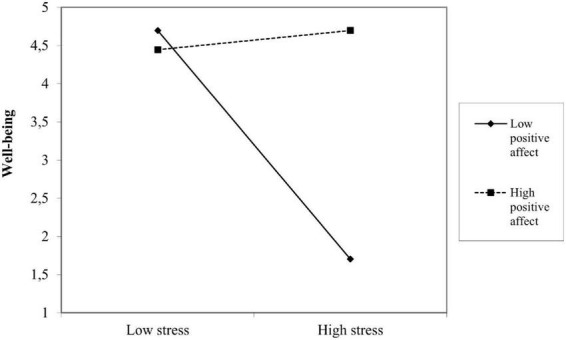
Moderation effect.

Finally, with Hypothesis 4 we proposed a moderation effect. We included positive affect as a moderator variable, such that entrepreneurs with higher positive affect would experience a decreased negative effect of perceived stress on their perceived wellbeing. Thus, we introduced the interaction term between perceived stress and positive affect. The results of the moderation analysis suggest that positive affect acts as a moderator in the relationship between perceived stress and wellbeing such that when positive affect is high, the negative effect of perceived stress on wellbeing is diminished, whereas for low levels of positive affect, the negative effect of perceived stress on perceived wellbeing is considerable. In statistical terms, the interaction term between perceived stress and positive affect has a positive and significant effect on entrepreneurs’ perceived wellbeing (β = 0.155, *p* = 0.029). The moderation effect is presented in Figure 3. Based on these results we have grounds to support Hypothesis 4. The model fit of the moderated-mediation model was appropriate with χ^2^ = 11.585; df = 11; *p* = 0.396; CFI = 0.996; RMSEA = 0.019; SRMR = 0.055. The *R*^2^ for entrepreneurial success was 0.211 and for subjective wellbeing *R*^2^ = 0.237. Statistical power was 1.0. With these results, we complement the literature, which shows that positive affect acts as a stress-buffering mechanism and directs entrepreneurs toward business efforts beyond what is immediately required (Foo et al., 2009) as well as to the achievement of better subjective wellbeing (Lyubomirsky et al., 2005; Lyubomirsky and Layous, 2013) and improved entrepreneurial success (Kaplan et al., 2009).

### Study 2: Qualitative data

Study 2 built on the quantitative data by examining the phenomena through in-depth personal interviews. We aimed to validate the legitimacy of the constructs used in our quantitative study and to further understand entrepreneurs’ perceptions and behaviors with respect to those constructs. Such research design has been proposed and explained by several scholars (Molina-Azorín et al., 2012; Creswell, 2015).

In the beginning, we wanted to hear how respondents described “success” as an entrepreneur. Several interviewees referred to success in financial indicators, such as revenues, profit, added value, cash flow, and export as measures of entrepreneurial success, which triangulates the proposition of the index measure used in Study 1. These are what our interviewees said (Interviewee #3): *“An entrepreneur needs to achieve some results, which are usually reflected in financial indicators, and to be successful such indicators need to be better than those of the competition”*; (Interviewee #7): *“For me, the entrepreneurial role models are those who have managed to sell their products abroad through their hard work and product development”*; and (Interviewee #10): *“First, I would say that a successful entrepreneur is the one who creates value-added, solves a real problem by providing a desirable solution and makes money from it.”*

Yet, quite some interviewees argued that to achieve such success and to fully understand success in an entrepreneurial context, we should take into consideration non-financial indicators of success, such as their work-life balance, satisfaction with work, satisfaction of their employees (employees’ personal growth, good relationships, positive organizational climate), customer satisfaction, and managing their company in a socially responsible way. This confirms the face validity of using job satisfaction to evaluate wellbeing in our empirical model in Study 1. This perspective can be supported by the following quotes (Interviewee #14): *“For me, growth of the firm is not the equivalent of success. For me, success in entrepreneurship is also when an entrepreneur likes the things s/he does at work and whether obtained results are enough for her/him. It is really important to be satisfied at work and with one’s life in general”*; and (Interviewee #8): *“The most important is personal satisfaction. Sometimes this sounds silly since we are in business for making money. But money is not the only goal. Satisfying other people’s needs and building relationships is the key. Being successful means that you attain personal satisfaction and happiness through attaining an entrepreneurial opportunity, which are also expressed toward others. A successful entrepreneur is a balanced entrepreneur.”* Drawing from our qualitative data, we validated our initial assumption that non-financial measures of success are salient to entrepreneurs; in our research, this is captured by entrepreneurs’ job satisfaction construct.

The next round of questions revolved around stress and coping with the daily challenges of entrepreneurship. The interviewees explained that stress was an integral part of entrepreneurship, i.e., (Interviewee #9): *‘Stress in entrepreneurship is an everyday issue. But, in business, you shouldn’t take things too personally. You should never get personally involved because you can get sick. You must know how to disconnect as well when as how to rest and relax*. Experiencing high levels of stress could put some burdens on achieving satisfaction and firm success, i.e., (Interviewee #7): *“If you are not able to cope with stress or eliminate it, you get in trouble*,” while on the other hand (Interviewee #6): *“The less stressed you are, the easier it is to work.”*

Thus, in the final part of the interviews, we asked respondents about their work affective experiences and ask them to relate such experiences to work-related stress and wellbeing and satisfaction. The interviewees agreed that when they experienced joy and enthusiasm at work, they felt less stress, were more satisfied and performed better at work. When under stress, experiencing positive affective emotions can represent viable coping mechanisms alongside having quality sleep, being physically active, and having a work-life balance. Positive affect promotes entrepreneurs’ confidence, action, and alertness. Interviewee #7 provided an illustration of this idea: *“I cope with stress by being resilient and in a good mood. When I experience positive emotions, I do my job better. If I do my job well, I am satisfied. This is a success for me. What is important in the end is how you feel about your successes.”* On the other hand, experiencing positive emotions less frequently represents saddles on achieving satisfaction (Interviewee #6): *“Currently, I experience so much stress and uncertainties, that I cannot enjoy myself. I am worried, rather than enthusiastic, so when I will pay off all the debt, I will be able to enjoy it. When I repay my debts, I will be able to enjoy more.”*

Our qualitative study provides insights into entrepreneurs’ perceptions of experiencing success and the major determinants of success in entrepreneurship. By interpreting the narratives of the interviewed entrepreneurs and exploring the phenomenon of success in entrepreneurship, we found that entrepreneurs perceived their success through the financial aspect and through the lenses of non-financial success indicators, such as feeling satisfied and experiencing overall wellbeing.

## Discussion

With this study, we propose a conceptual model of the relationships connecting stress to the level of perceived wellbeing and an important outcome—firm success as viewed by an entrepreneur. We have developed conceptual arguments to explain why the pathways between stress and entrepreneurial success are direct and mediated by the level of wellbeing. In addition, we have provided a boundary condition for the effects of stress on the outcome variable by explaining how positive affect moderates the relationship between stress and wellbeing such that the indirect effects through the perceived level of wellbeing are weaker for higher levels of positive affect. We find that positive affect is an important and positive moderator that makes the mediated relationship between stress and entrepreneurial success less negative. As previous research showed that positive affect motivates entrepreneurs to focus on subjective wellbeing (Lyubomirsky et al., 2005; Lyubomirsky and Layous, 2013) and improved performance (Kaplan et al., 2009), we show positive affect diminishes the effects of stress on the level of entrepreneurial success. Thus, we provide further support to the literature highlighting the importance of positive affect in entrepreneurship, for example, in influencing key individual-level outcomes, such as creativity (Baron and Tang, 2011), opportunity recognition, evaluation, and effective decision-making (Baron et al., 2012).

### Scholarly and practical implications

Our first contribution is to the literature exploring the work-related stress of entrepreneurs and wellbeing. We find that perceived wellbeing partially mediates the relationship between perceived stress and entrepreneurs’ wellbeing (indirect effect: β = −0.083, *p* = 0.005). As the literature suggests (see for example Baron et al., 2016), entrepreneurs experience stress regularly, independent of the performance level of a firm. This stress is due to the adverse conditions in which they fulfill their occupational roles. We also show that positive affect buffers potentially detrimental effects of experienced stress on subjectively perceived wellbeing as well as entrepreneurial success. With our study, we respond to an unanswered question posed by this stream of research: How can entrepreneurs better manage their stress without damaging their health? In addressing this question, we integrate theory and evidence from medical and organizational studies to propose a model in which the direct effects of stress on perceived success are mediated by the entrepreneurs’ wellbeing.

Our second contribution is to the extant body of literature on positive affect in entrepreneurship (Baron, 2008; Baron et al., 2011; Cardon et al., 2012). Our findings emphasize the importance of positive affect in impacting entrepreneurs’ wellbeing. Specifically, we show that when positive affect is high, the negative effect of perceived stress on wellbeing is diminished, whereas for low levels of positive affect the negative effect of perceived stress on perceived wellbeing is substantial (β = 0.155, *p* = 0.029). Our research suggests that entrepreneurs who have high positive affect may be less affected by the level of experienced stress because positive affect broadens and builds individuals’ thought-action repertoires, prompting them to pursue a wider range of thoughts and actions. In sum, we expand the literature in this area by exploring the intervening mechanisms of positive affect in entrepreneurs’ experience of wellbeing.

Our findings also have some implications for practice. The role of regular exercise is unprecedented in enabling entrepreneurs to effectively cope with the occupational demands of their workplace. Although regular exercise takes time and effort, entrepreneurs may perhaps have more flexibility and freedom to work out whenever they want, compared to other occupations. Given the high odds of experiencing business failure or loss of control throughout events at some point in one’s entrepreneurial career (Everett and Watson, 1998), regular exercise can enhance entrepreneurs’ feelings of control over their life. Indeed, rich research evidence tells us that individuals who exercise regularly will improve their health (Nelson et al., 2007), assuming all else remains the same. Regular exercise makes people more creative and productive (Gremeaux et al., 2012), since exercise increases cognitive functioning, boosts physical energy, and reduces fatigue (Puetz et al., 2008). Finally, as we have argued, regular exercise significantly reduces stress, which is a prevalent side effect of running a venture. Hence, we recommend that entrepreneurs overcome their reluctance to engage in regular exercise because of the time or energy costs. Research has shown that entrepreneurs should engage in regular physical exercise, as the positive effects of regular exercise more than compensate for the time invested in exercise.

In addition, our findings also suggest that positive affect is a source of personal strength (Baron et al., 2016), reducing the effects of stress on the level of perceived entrepreneurial success. This suggests that entrepreneurs need to pay attention to their experienced emotions and regulate them to reap the positive benefits by balancing their professional and personal lives. Specifically, beneficial effects of positive affect can be expected when individuals can identify and categorize it, which suggests that entrepreneurs can be coached to be more observant of its occurrence (Frijda, 1993). In the words of the founder of Eventbrite, “Finding balance is an ongoing challenge, which requires constant attention and dedication. My life is extremely binary—my passion is in Eventbrite and my love is in my family. The nature of business today is that the lines of ‘work’ and ‘life’ are a little more blurred” (Rampton, 2019). Finally, our findings suggest that entrepreneurship education should redirect some of its focus away from the cognitive and emotional aspects of entrepreneurship and toward the role of physical fitness.

### Limitations and future research opportunities

Like in any research, there are some limitations to this study, which present potential opportunities for future research. First, this study was designed to explore the effects of entrepreneurs’ perceived stress on entrepreneurial success using a mixed methods approach. The data for the quantitative portion of the study were collected using a survey among a random anonymous sample of founders through a cross-sectional design. The latter technique introduces potential problems of causality and common method variance. To address the potential problem of causality, we made sure to hypothesize associations rather than causations when theoretically building our hypotheses and interpreting our empirical findings. In the future, a longitudinal design could help to establish the causal effects of stress on entrepreneurial outcomes. To evaluate the possibility of common method variance in the empirical data, we applied procedural and statistical remedies *ex ante* and *ex post* data collection and data analysis. Although we are not able to rule out the possibility of common method variance with complete certainty, we can claim with a high level of confidence that common method bias was not a threat to our data, based on the procedures we followed. On the other hand, future researchers could test the model by compiling an empirical sample using a combination of self-report data and objective data, for example, by including objective measures (i.e., financial) of entrepreneurial success. Because we assured the anonymity of our respondents, we were not able to link their responses to financial indicators of their ventures. Future researchers could also collect alternative indicators of independent variables in the models, such as objectively measuring entrepreneurs’ stress levels through biological markers, such as salivary cortisol levels. In addition, we would like to emphasize that additional statistical tests performed do not suggest that multicollinearity poses a threat to our findings.

In addition, some may argue that a reverse causation from entrepreneurial success to stress might take place. Even more, there can be also a possibility of the simultaneity effect between the two constructs. To test the above-mentioned potential effects, we tested alternative empirical models. First, we checked for simultaneity—the reverse loop effects between stress and entrepreneurial success. The results of model testing yielded the following result: stress was negatively related to entrepreneurial success (β = −0.579, *p* = 0.031), while entrepreneurial success was not significantly related to stress (β = 0.262, *p* = 0.181). Second, we checked an alternative model of reverse causation in which entrepreneurial success was modeled as an exogenous variable, wellbeing as a mediator and stress as an endogenous variable. Positive affect acted as a moderator in the relationship between wellbeing and stress. The results showed a significant decrease in the model fit (original model: CFI = 0.996, SRMR = 0.056, RMSEA = 0.019; reverse causation model: CFI = 0.941, SRMR = 0.080, RMSEA = 0.075). In addition, the model displayed no moderation effect, while the relationship between entrepreneurial success and stress was statistically significant and negative (β = −0.149, *p* = 0.036). Yet, such reverse causations are better analyzed in a longitudinal setting and are beyond the scope of this research.

Finally, we need to note that the gender structure of the datasets for the two studies differed. Study 1 (the quantitative study) included 53% of female entrepreneurs, there were only 13% of female entrepreneurs included in Study 2 (i.e., the qualitative study). Although the dataset for Study 1 was comprised by randomly selected micro and small firms taken from the database Bizi.si, the survey was answered by a higher than usual percent of female entrepreneurs. This might suggest that the research topic has been of greater interest to female than to male entrepreneurs, or that some other random effect might have taken place. In Study 2 we paid particular attention to include a higher percentage of male entrepreneurs (i.e., 73%) to better account for gender structure of founders of micro and small firms in Slovenia.

In our research, we applied a mixed method explanatory sequential research design (Molina-Azorín et al., 2012; Creswell, 2015). Such research design has several advantages and could be used in future business-related research. First, it helps researchers better understand the quantified relationships among constructs under study by qualitatively interpreting them with the help of interviews’ analyses. Second, it adds value to the research results which are gathered through quantitative and qualitative methods by providing more substance and proof. Yet, such research requires a double amount of work in comparison to either solo-quantitative research setting or a solo-qualitative research setting. We also must note that reporting both, quantitative and qualitative studies, requires a lot of space in a manuscript, providing challenges for scholars to report as much in a limited space.

## Data availability statement

The raw data supporting the conclusions of this article will be made available by the authors upon reasonable request.

## Ethics statement

Ethical review and approval was not required for the study on human participants in accordance with the local legislation and institutional requirements. Written informed consent from the patients/participants or patients/participants legal guardian/next of kin was not required to participate in this study in accordance with the national legislation and the institutional requirements.

## Author contributions

MD: conceptualization, literature review, writing, and data collection. ASG: conceptualization, writing, data collection, and empirical analysis. Both authors contributed to the article and approved the submitted version.

## Appendix

**TABLE A1:** Final scale items and standardized loadings based on the CFA.

Scales	Loading
**Perceived stress** (Cohen et al., 1983) (never/very often)	
In the last month, how often have you been upset because of something that happened unexpectedly?	0.656
In the last month, how often have you felt that you were unable to control the important things in your life?	0.789
In the last month, how often have you felt confident about your ability to handle your problems? (reverse scored)	0.709
In the last month, how often have you felt that things were going your way? (reverse scored)	0.733
In the last month, how often have you found that you could not cope with all the things that you had to do?	0.546
In the last month, how often have you been able to control irritations in your life? (reverse scored)	0.577
In the last month, how often have you felt that you were on top of things? (reverse scored)	0.660
In the last month, how often have you been angered because of things that happened that were outside of your control?	0.503
In the last month, how often have you felt difficulties were piling up so high that you could not overcome them?	0.753
**Wellbeing/job satisfaction** (Judge et al., 1998) (strongly disagree/strongly agree)	
I feel fairly satisfied with my present job.	0.756
Most days I am enthusiastic about my work.	0.918
I find real enjoyment in my work.	0.717
**Positive affect** (Watson et al., 1988) (strongly disagree/strongly agree)	
Strong	0.507
Enthusiastic	0.785
Proud	0.674
Attentive	0.644
Interested	0.708
Determined	0.636
**Entrepreneurial success** (index)	
Firm success in the last 3 years in terms of revenue growth and market share growth (1 = much worse than competitors, 5 = much better than competitors) relative to the geographical position of the majority of customers (1 = mostly foreign markets, 2 = mostly national market, 3 = mostly local market).	/
